# Poly(ethylene glycol)-Engrafted Graphene Oxide for Gene Delivery and Nucleic Acid Amplification

**DOI:** 10.3390/ma16237434

**Published:** 2023-11-29

**Authors:** Khushbu Chauhan, Jin Woo, Woong Jung, Dong-Eun Kim

**Affiliations:** 1 Department of Bioscience and Biotechnology, Konkuk University, 120 Neundong-ro, Seoul 05029, Republic of Korea; 2 Department of Emergency Medicine, Kyung Hee University College of Medicine, Kyung Hee University Hospital at Gangdong, Seoul 05278, Republic of Koreaondali77@khu.ac.kr (W.J.)

**Keywords:** graphene oxide, biocompatibility, dispersibility, cytotoxicity, polyethylene glycol, gene/drug delivery, nucleic acid amplification

## Abstract

Graphene oxide (GO) is an oxidized form of graphene accommodating various oxygen-containing functional groups such as hydroxyl, epoxy, and carboxyl groups on its surface. GO has been extensively utilized in various biomedical applications including the delivery of biomolecules and the development of biosensors owing to its beneficial properties such as high surface area, nucleic acid adsorption, and fluorescence quenching through fluorescence resonance energy transfer (FRET). However, despite these favorable properties, the direct utilization of GO in these applications is often limited by low dispersibility in a physiological medium, cytotoxicity, low biocompatibility, and a strong binding affinity of nucleic acids to GO surface. The large surface area of GO and the presence of various functional groups on its surface make it highly amenable to facile surface modifications, offering scope for GO surface functionalization to overcome these limitations. When polyethylene glycol (PEG), which is a biocompatible polymer, is conjugated to GO, the PEGylated GO enhances the biocompatibility and dispersibility, reduces cytotoxicity, and allows controlled drug delivery with controllable binding affinity towards nucleic acid. PEG-engrafted GO retains the beneficial properties of GO while effectively addressing its limitations, rendering it suitable for various biomedical applications. In this review, we present the recent advancements of PEGylated GO in gene/drug delivery and the facilitation of nucleic acid amplification techniques, which aid in the development of therapeutic and diagnostic tools, respectively.

## 1. Introduction

Graphene sheets, characterized by their regular hexagonal arrangement of sp^2^ hybridized carbon atoms in the *xy*-plane, possess an almost negligible thickness along the *z*-plane, making them one of the most promising carbon allotropes [1]. The single layered carbon atoms in the two-dimensional honeycomb structure of graphene contribute to its exceptional attributes, such as high electronic mobility, mechanical elasticity, and high thermal conductivity [2,3,4]. However, the application of pristine graphene has encountered several limitations because of poor solubility [5,6], agglomeration in solution attributed to van ser Waals interaction [7], and challenging bottom-up synthesis [8].

**Figure 1:**
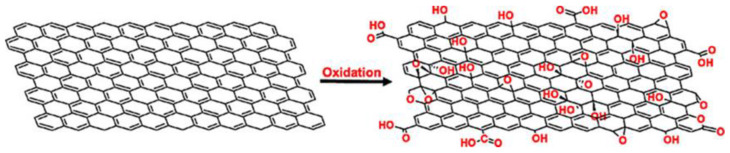
Oxidation of graphene to form graphene oxide. Reprinted from [19].

Graphene oxide (GO) is the chemically oxidized form of graphene (Figure 1) that is hydrophilic, industrially scalable, and cost effective with profound implications in various scientific and industrial realms including the biomedical industry [9,10,11]. GO demonstrates high hydrophilicity and improved dispersibility in water and polar organic solvents, owing to the presence of various oxygen-related functional groups such as carboxyl, hydroxyl, carbonyl, and epoxide groups [12,13,14,15]. Carboxylic groups are predominantly situated along the edges of GO, while epoxide and hydroxyl groups are distributed across the basal plane of GO [16,17,18,19].

**Figure 2:**
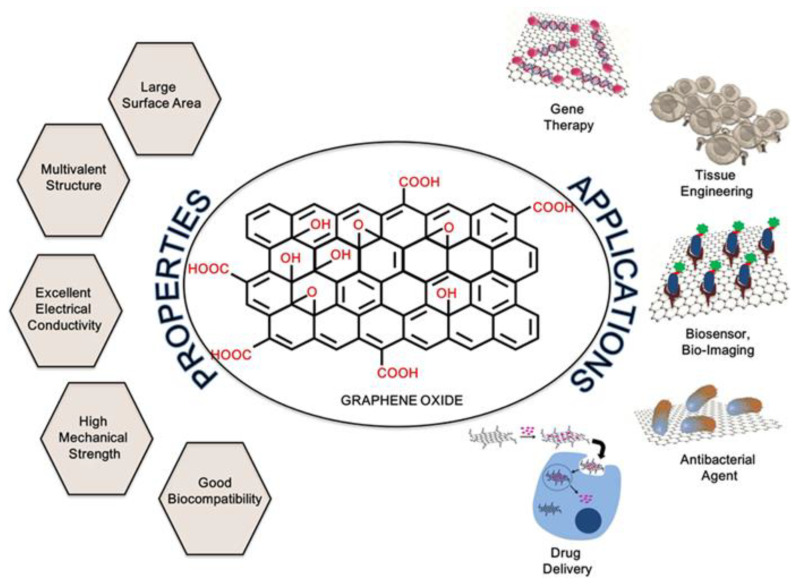
The properties of GO and its biomedical applications. Reprinted from [20].

GO possesses numerous intriguing characteristics that render it highly valuable in a variety of biomedical applications such as drug delivery, tissue engineering, bioimaging, and biosensor development (Figure 2). One of the most significant features is its high surface area, a result of its two-dimensional structure combined with the presence of oxygen-based functional groups. The high surface area of GO allows for efficient loading of drugs, and its ability to release them in a controlled manner has the potential to enhance treatment outcomes [20,21,22,23]. Additionally, its suitability for surface functionalization due to the presence of various functional groups allows for tailored modification, enabling researchers to fine-tune its characteristics to suit specific requirements [24,25]. This adaptability is invaluable in the development of tailored drug delivery systems and diagnostic tools. GO’s moderate electrical conductivity has been harnessed for the development of electro-responsive drug delivery systems, which enable on-demand release of drugs in response to external stimuli [26,27]. Also, GO exhibits a strong interaction with a wide range of small molecules and macromolecules (such as drugs, ions, proteins, metals, and cells) via π–π stacking, hydrophobic interactions, and hydrogen bonding [28,29,30]. GO selectively absorbs single-stranded nucleic acids through π–π stacking and hydrogen bonding, while double-stranded DNA has minimal adsorption due to the folded structure of DNA [31,32,33], and GO also exhibits fluorescence quenching ability through fluorescence resonance energy transfer which forms the basis of GO-based biosensor development [34,35,36,37,38].

**Figure 3:**
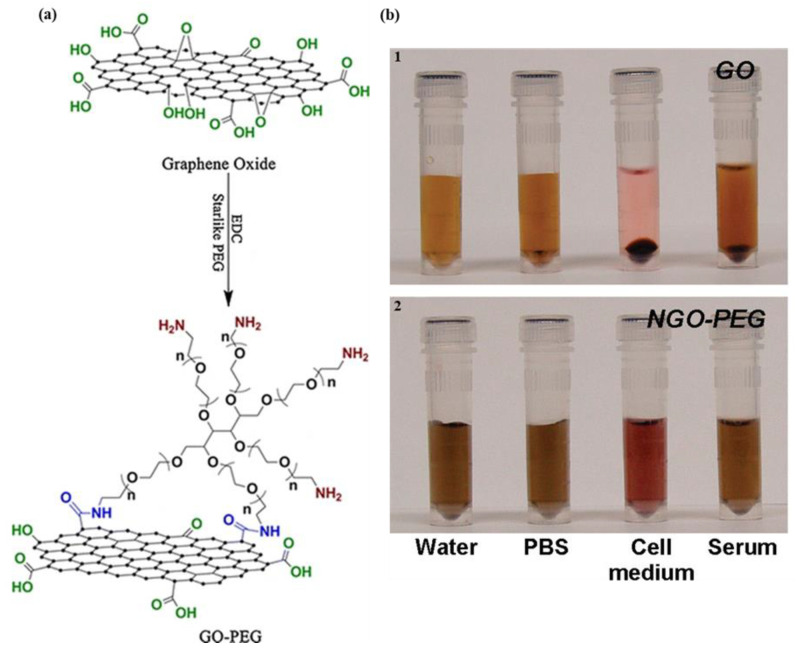
PEGylated GO. (**a**) Synthesis and structure of PEGylated GO from GO. Reprinted with permission from [47]. Copyright © 2014, American Chemical Society. (**b**) Enhanced solubility of PEGylated GO in comparison to GO. Reprinted with permission from [41]. Copyright © 2008, American Chemical Society.

Despite having such remarkable properties, GO harbors some inherent constraints when employed in various biomedical applications. These limitations include its potential to trigger an immune response due to its foreign nature in the body, non-specific binding with biomolecules, propensity for poor aqueous stability causing aggregation, a relatively brief circulation time in the bloodstream, toxicity, and limitations in drug loading capacity [39,40,41,42,43]. Polyethylene glycol (PEG) is a biocompatible polymer which is being utilized for various biotechnological applications. Owing to the large surface area and various oxygen-containing functionalities on its surface, GO provides the advantage of easy surface modification (Figure 3a). The functionalization of the GO surface with PEG can effectively overcome these challenges while retaining the beneficial properties of GO. The conjugation of PEG chains to GO demonstrated enhanced biocompatibility, minimized the risk of immune responses, reduced nonspecific binding, improved solubility, and prevented aggregation in various physiological media (Figure 3b). Moreover, PEG-GO extends the circulation time in the bloodstream, allowing for a more effective accumulation at the desired sites, thereby improving therapeutic outcomes [41,44,45,46]. Thus, PEGylated GO serves as a crucial advancement, rendering GO a more suitable and effective material for various biomedical applications. In this review article, we present the latest progressions involving the utilization of PEG-modified GO in the realms of gene and drug delivery, and the enhancement of nucleic acid amplification technologies.

## 2. Functionalization of GO

The facile surface modification of GO is attributed to its diverse oxygen containing functional groups, high surface area, and hydrophilic characteristics. This ease of surface modification enhances solubility, stability, and biocompatibility, making GO indispensable for a wide range of biomedical applications. The functionalization of GO with various biomolecules is mainly carried out via covalent conjugation and non-covalent attachment. The presence of hydroxy, epoxy, and carboxy entities on GO surfaces allows covalent functionalization through various reactions such as esterification, click chemistry, and amidation [3,48,49,50]. In addition, non-covalent functionalization of GO is carried out via reactions using hydrogen bonding, electrostatic interactions, and π-π interactions [51,52,53]. Biocompatible polymer PEG can be conjugated or attached to GO via covalent or non-covalent functionalization, respectively, for various applications (Table 1).

Our group synthesized poly(ethylene glycol)-engrafted nanosized graphene oxide (PEG-nGO) for the enhancement of nucleic acid amplification and nucleic acid delivery [54,63,64]. Briefly, PEG-nGO was synthesized by adding NaOH and chloroacetic acid to the sonicated GO (conversion of −OH groups to −COOH groups). The resulting HOOC-nGO was repeatedly rinsed with water and purified by a 0.2 μm filter membrane. A 6-armed PEG-amine and *N*-3-(dimethylamino)propyl-*N*′-ethylcarbodiimide hydrochloride (EDC) was added to the reaction mixture and stirred for 12 h. The mixture was centrifuged in phosphate buffer saline, saving the supernatant containing PEG-nGO. Characterization of PEG-nGO was carried out by atomic force microscopy which showed a smaller particle size of PEG-nGO (200 nm) than GO (400–1000 nm) and increased thickness (4–5 nm) as compared to GO (1.2–1.6n nm). PEGylation of GO was also confirmed by Fourier transform infrared spectroscopy where PEG-nGO showed methylene (2800 cm^−1^) and amide carbonyl bands (1650 cm^−1^), suggesting the conjugation of PEG to the GO surface [54,63].

## 3. PEG-Engrafted GO for Biomolecule Delivery

The development of efficient nano delivery systems is crucial for nanomedicine and modern therapeutic applications. Different materials such as micelle, liposomes, metal oxides, inorganic nanomaterials, and various biopolymers have been employed for biomolecule delivery but are often limited by low efficacy, poor solubility, non-specific targeting, cytotoxicity, hampered retention time, metabolization, etc. [65,66]. GO has the potential to overcome these limitations as it exhibits numerous advantageous features such as high loading capacity, various functional groups on the surface, ease of surface functionalization, fluorescence quenching, and the ability to adsorb a wide range of biomolecules on its surface, rendering it an excellent candidate for therapeutic applications. However, the recent advancements in GO-based biomolecule delivery systems are often challenged by low solubility, reduced permeability, toxicity, nonspecific targeting, and rapid drug metabolism. Due to its facile surface modification, GO can be functionalized with diverse biocompatible polymers, such as PEG, offering biocompatibility, superior aqueous solubility, low toxicity, and prolonged drug circulation. The biomedical applications of biocompatible PEG-conjugated GO have expanded its utility in therapeutics, employing biocompatible GO derivatives as carriers for gene and drug delivery.

### 3.1. Nucleic Acid Delivery

**Figure 4:**
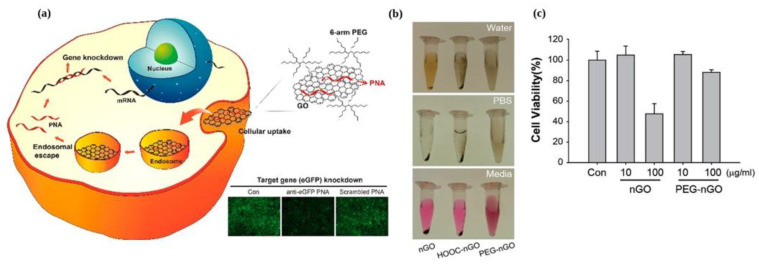
PEG-nGO as a biocompatible carrier for PNA delivery. (**a**) Schematic representation showing specific gene knockdown via PEG-nGO mediated delivery. (**b**) Enhanced solubility of PEG-nGO and various physiological media. (**c**) Comparison of cytotoxicity of nGO and PEG-nGO in small lung cancer cells. Reprinted with permission from [54]. Copyright © 2018, American Chemical Society.

Owing to its ability to bind single-stranded nucleic acids and fluorescence quenching near its surface, GO has emerged as a promising vehicle for gene delivery. Our group utilized PEGylated nanosized GO (PEG-nGO) as a biocompatible carrier with excellent solubility and low toxicity to deliver antisense peptide nucleic acid (PNA) against EGFR mutant RNA into lung cancer cells (Figure 4) [54]. The ability of GO to absorb ssDNA on its surface via hydrophobic interaction and hydrogen bonding allowed the neutral PNA to easily absorb onto the PEG-nGO surface using similar interactions. This adsorbed PNA was released by introducing complementary RNA or under low pH conditions. The PNA/PEG-nGO complex was internalized via endocytosis, where PEG-nGO retained in endosomes and the PNA was released into the cytosol under low pH conditions. Importantly, PEG-nGO demonstrated no cytotoxicity and did not induce autophagic reactions in the cells, making it a promising biocompatible gene delivery carrier.

Zhang et al. focused on the development of PEGylated reduced GO (PEG-RGO) as a potential delivery system for ssRNA molecules [55]. The research involved synthesizing PEG-RGO through a combination of PEGylation and reduction processes and then comparing its ssRNA loading and delivery capabilities with those of PEGylated GO (PEG-GO). The findings revealed that PEG-RGO outperformed PEG-GO in terms of its efficiency in loading and delivering ssRNA to HeLa cells. Computational simulations support these experimental results, showing stronger π–π stacking interactions between ssRNA and RGO as compared to GO. This study offers a promising approach for the biocompatible and efficient delivery of ssRNA and suggests that PEG-RGO may serve as a valuable nano vector for delivering various biomolecules in biomedical therapeutics.

Imani et al. presented a comprehensive investigation of a dual-functionalized GO-based nanocarrier with the conjugation of aminated-polyethylene glycol (PEG-diamine) and octa-arginine (R8) for the intracellular delivery of nucleic acids in the treatment of cancer [56]. This study focused on optimizing a nanocarrier capable of delivering both siRNA and plasmid DNA, a unique feature that differentiates it from previous studies. The authors optimized the PEG: R8 molar ratio to achieve a stable, positively charged nanocarrier. The optimized nanocarrier, GPPo, showed effective transfection in breast cancer cell lines, MCF-7 and MDA-MB 231, outperforming a commercial transfection reagent, HiPerFect^®^ QIAGEN Inc. (Toronto, ON, Canada). Furthermore, functional gene delivery was confirmed through c-Myc protein knockdown and EGFP gene expression, underscoring the potential of this nanocarrier. The study’s significant contributions lie in its dual functionality and efficient nucleic acid delivery capabilities. The authors acknowledge the need for further improvement, particularly in enhancing targetability and endosomal escape, which are crucial for clinical applications.

**Figure 5:**
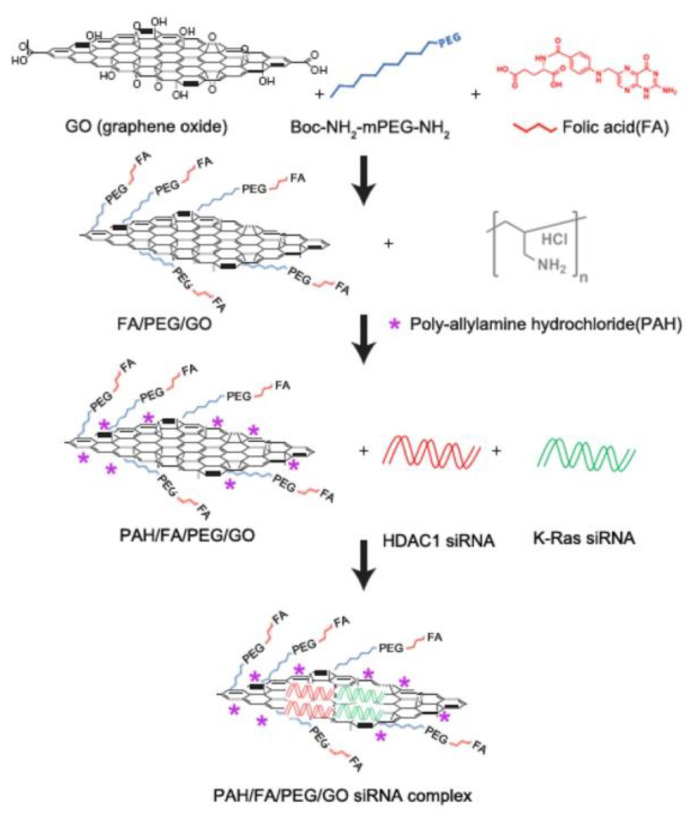
Schematic overview of the FA/PEG/GO synthesis and gene loading process using engineered GO-based nanocarriers. Reprinted from [67].

Yin et al. investigated the application of PEGylated GO nanosheets for combined photothermal and gene therapy in the context of pancreatic cancer [67]. As shown in Figure 5, the authors demonstrated the potential of multi-functionalized monolayer GO as a gene delivery platform to co-deliver HDAC1 and K-Ras siRNAs (small interfering RNAs targeting the HDAC1 gene and the G12C mutant K-Ras gene, respectively) to specifically target pancreatic cancer cells. Their findings unveiled a promising dual gene silencing strategy, resulting in the inactivation of both HDAC1 and K-Ras genes, thereby inducing apoptosis, halting proliferation, and causing cell cycle arrest in MIA PaCa-2 cells. The combination of gene silencing and near-infrared light thermotherapy exhibited significant anticancer efficacy, with an over 80% reduction in in vivo tumor growth. Furthermore, the study demonstrated the biodegradability of GO in mouse models with minimal side effects, paving the way for future applications in gene therapy for pancreatic adenocarcinoma.

The study conducted by Feng et al. explored the development of a novel nanomedicine platform by GO with PEG and polyethylenimine (PEI) to create a dual-polymer-functionalized nano-GO conjugate, referred to as NGO-PEG-PEI [57]. This hybrid nanostructure offers a solution to some of the challenges associated with gene delivery. One of the noteworthy contributions of this research was the improved physiological stability and gene transfection efficiency achieved with NGO-PEG-PEI, especially in the presence of a serum-containing cell medium. Unlike free PEI or GO-PEI complexes, the NGO-PEG-PEI exhibited reduced cytotoxicity and enhanced gene transfection efficiency. Furthermore, the study demonstrated enhanced cellular uptake of NGO-PEG-PEI under low power NIR laser irradiation. It harnessed the near-infrared (NIR) optical absorbance of NGO. This study was the first to use photothermally enhanced intracellular trafficking of nanocarriers for light-controllable gene delivery. This work also encouraged further explorations of functionalized nano-GO as a photo-controllable nanovector for combined photothermal and gene therapies. The significant outcomes of the research included the remarkable enhancement in plasmid DNA transfection efficiency and the successful delivery of small interfering RNA (siRNA) under NIR light control. The study thus pioneered the use of photothermally enhanced intracellular trafficking of nanocarriers for light-controllable gene delivery, marking an advancement from previous approaches that relied on the heat-triggered intracellular release of cargo molecules from nano-carriers.

**Figure 6:**
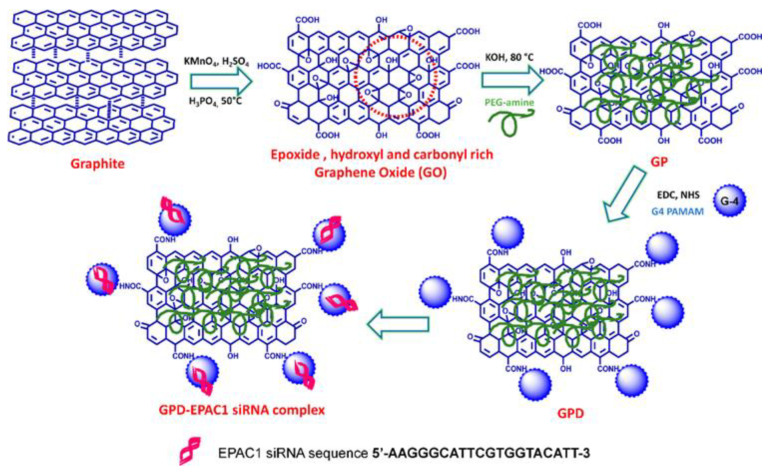
Synthetic scheme for the preparation of a GO-based nanocarrier to load siRNA (GP, PEGylated GO; GPD, PAMAM functionalized GP). Reprinted with permission from [68]. Copyright © 2018, American Chemical Society.

A study by Yadav et al. explored the covalent tethering of poly(amidoamine) (PAMAM) and PEG-modified GO to create a hybrid vector known as GPD (Figure 6), in which the integration of these three components served multiple purposes in improving siRNA delivery [68]. In particular, the covalent linkage of PEG to the GO-PAMAM structure enhanced the stability of aqueous dispersion, a crucial factor for clinical application. This stability at physiological pH was achieved without requiring extensive purification. One key advantage of PEG in the GPD structure was its ability to reduce the cytotoxicity associated with PAMAM and protect siRNA from enzymatic cleavage. PAMAM, on the other hand, provided amine groups for siRNA binding and facilitated cellular entry, thus enhancing the delivery of siRNA into target cells. Additionally, GO served as a versatile conjugation platform, enabled the loading of siRNA with reduced toxicity-enhanced dispersion stability of PAMAM. These multiple functionalities contributed to the improved performance of the GPD vector. The study demonstrated that GPD was highly efficient in siRNA delivery with low cytotoxicity, enhanced cellular uptake, and effective gene silencing. Moreover, GPD outperformed other commonly used nonviral vectors like PAMAM and Lipofectamine 2000, particularly in terms of transfection efficiency, metastasis prevention, and inhibition of cell invasion. The pH-responsive siRNA release further indicated the controlled and sustained release of siRNA under acidic conditions, offering mechanistic insights into the unloading of siRNA from the vector. This study provided a promising approach for designing more effective therapeutic vectors for gene-based antitumor therapy.

The study conducted by Szénási et al. expanded the previous findings that superoxide dismutase 1 (SOD1) overexpression in ovarian cancer leads to platinum resistance due to its role in conferring oxidative stress resistance against platinum compounds [69]. Platinum resistance is a major challenge in the treatment of ovarian cancer, contributing to a substantial number of annual deaths globally. Their study investigated the potential of targeting SOD1 via RNA interference (RNAi) using PEGylated GO nanoparticles in platinum-resistant ovarian cancer. The researchers developed a siRNA delivery platform with PEGylated graphene oxide (GOPEI-mPEG) nanoparticles complexed with SOD1 siRNA. In vitro experiments demonstrated that inhibiting SOD1 through small-molecule inhibitors or RNAi increased cisplatin sensitivity. The study highlighted the potential therapeutic use of RNAi-mediated SOD1 targeting as a chemosensitizer for platinum-resistant ovarian cancers. Despite promising results, the study also identified serious challenges, particularly related to nanoparticle-mediated toxicity and the potential counteractive effect of nanoparticles on SOD1 knockdown. The authors noted the need for further investigation using alternative drug delivery platforms, such as lipid nanoparticles (LNPs), and different xenograft models. These findings contributed to the ongoing efforts to develop innovative strategies to overcome platinum resistance in ovarian cancer.

### 3.2. Drug and Protein Delivery

PEGylated GO enhances the pharmacokinetics of drug delivery systems due to its high loading capacity and low toxicity. The attachment of PEG chains increases the circulation time of GO in the bloodstream, effectively extending the duration during which it can accumulate at target tissues. This leads to a higher concentration of drug delivery at the desired sites, significantly improving the efficacy of therapeutic treatments.

PEGylated nano GO (NGO-PEG) has been utilized by Lie et al. for delivering water-insoluble anticancer drugs into human colon cancer cells [41]. The study demonstrated complexing of NGO-PEG with the water-insoluble drug SN38, an analogue of camptothecin used in colon cancer treatment, through noncovalent interactions. Upon delivery via endocytosis, this complex improved the water solubility and stability of the drug, displaying unhindered potency of SN38 as an anticancer agent within the cancer cells. Notably, the NGO-PEG as a drug delivery carrier did not exhibit any cytotoxicity or induce apoptotic cell death, indicating no side effects attributed to PEGylated nanographene oxide.

**Figure 7:**
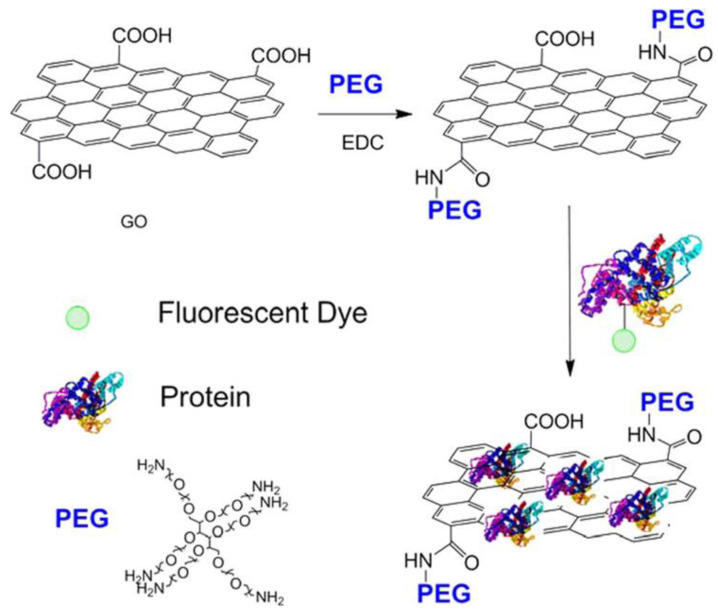
Schematic representation showing the formation of the GO-PEG/BSA-FITC complex. Reprinted with permission from [70]. Copyright © 2012, American Chemical Society.

The study by Shen et al. functionalized GO with an amine-terminated 6-armed PEG (Figure 7), enhancing its physiological stability and biocompatibility [70]. This delivery system successfully loaded different proteins onto PEG-grafted GO (GO-PEG) through noncovalent interactions, facilitating the efficient delivery of proteins to the cytoplasm while protecting them from enzymatic hydrolysis. The significant finding of this research was that the proteins delivered by GO-PEG retained their biological activity, allowing them to regulate cell fate. The study demonstrated this by inducing cell death with ribonuclease A and cell growth with protein kinase A, highlighting the potential therapeutic applications for this protein delivery system.

**Figure 8:**
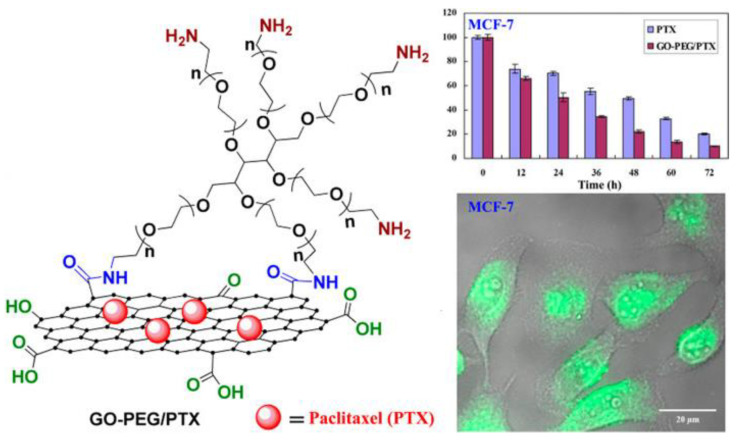
Schematic representation showing the formation of a GO-PEG-PTX nanoscale drug delivery system. Reprinted with permission from [47]. Copyright © 2015, American Chemical Society.

The study by Xu et al. discussed the use of PEGylated GO as a nanocarrier for delivering the chemotherapy drug Paclitaxel (PTX) (Figure 8) [47]. PTX, while effective, suffers from low water solubility, poor bioavailability, and drug resistance issues. This study proposed a novel drug delivery system based on GO to enhance the effectiveness of PTX in cancer therapy. The study involved a multi-step process where PTX was chemically modified to make it more compatible with GO, creating a PTX-terminated PEG. Subsequently, this modified PTX was attached to biocompatible 6-armed starlike PEG to produce GO-PEG-PTX. The successful synthesis of GO-PEG-PTX was confirmed through various analyses, including UV–vis spectroscopy, atomic force microscopy, and thermogravimetric analysis. The results of this study showed that GO-PEG-PTX was quickly taken up by cancer cells, particularly A549 and MCF-7 cells, indicating its potential as an effective drug delivery system for PTX.

Miao et al. investigated the safety and potential of PEG-grafted GO (pGO) nanosheets as a versatile nanocarrier for the co-delivery of photosensitizers and anticancer agents [58]. Synthesis of pGO was carried out, and in vitro and in vivo toxicity were checked. Intriguingly, pGO demonstrated superior safety in vivo, as evidenced by 100% survival rates among mice treated with pGO nanosheets compared to 100% fatality in the GO-treated group. The study then explored the enhanced cellular delivery of the photosensitizer chlorin e6 (Ce6) when loaded onto pGO nanophysisorplexes. Co-loading Ce6 with doxorubicin (Dox) in a molar ratio of 1:2 resulted in the highest synergism, highlighting the potential of this co-delivery system. In tumor-bearing mice, Ce6/Dox/pGO exhibited superior photodynamic anticancer effects compared to other groups, leading to substantial disruption of tumor nuclei. These findings suggested that pGO nanosheets offer enhanced in vivo safety over GO and have the potential to improve tumor tissue distribution and photodynamic anticancer effects when co-delivering chemotherapeutics like Dox with photosensitizers.

**Figure 9:**
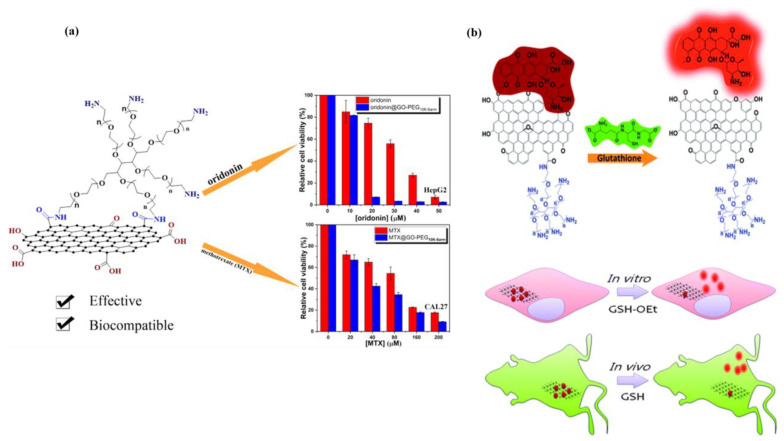
(**a**) PEGylated GO-based nanoscale drug delivery system for oridonin and methotrexate delivery. Reprinted with permission from [59]. Copyright © 2019, American Chemical Society. (**b**) Real-time detection of the drug release by monitoring the recovery of fluorescence quenching of Dox on PEG-GO after the external triggering of GSH-OEt in vitro and GSH in vivo. Reprinted from [71].

The study performed by Chai et al. explored the potential of a novel drug delivery platform involving PEGylated GO (GO-PEG_10K-6arm_) for the delivery of hydrophobic anticancer drugs, specifically oridonin and methotrexate (MTX) (Figure 9a) [59]. GO-PEG_10K-6arm_ was prepared by conjugating 6-armed PEG to the surface of GO via an amidation reaction. This modification enhanced the water solubility and biocompatibility of GO, rendering it a suitable nanocarrier for drug delivery. The nanosized GO-PEG_10K-6arm_ platform exhibited low toxicity to both normal and tumor cells. Oridonin@GO-PEG10K-6arm and MTX@GOPEG_10K-6arm_ nanocomplexes were formed through π–π stacking and hydrophobic interactions, which enabled the rapid uptake of these complexes into tumor cells. The study found that both nanocomplexes displayed high cytotoxicity to various tumor cell lines compared to free drugs. This demonstrated the potential of GO-PEG_10K-6arm_ as an effective nanoscale drug delivery system, enhancing the solubility and bioavailability of hydrophobic anticancer drugs.

In another study carried out by Dembereldorj et al., the authors explored a novel spatiotemporal anticancer drug release platform involving PEGylated GO (PEG-GO) and its response to glutathione (GSH) triggers (Figure 9b) [71]. The authors employed live-cell fluorescence imaging, a powerful technique for non-invasive and real-time assessment of dynamic interactions. Their study demonstrated a comprehensive investigation of intracellular drug release from PEG-GO triggered by GSH, both in vitro and in vivo. Additionally, the study demonstrated the feasibility of in vivo drug release monitoring through fluorescence imaging in living mice following GSH treatment. This innovative approach, facilitated by label-free fluorescence quenching measurements, offers a promising avenue for real-time tracking of drug release from PEG-GO in both cellular and animal models.

A study by Pei et al. explored the use of PEGylated nano-GO (pGO) as a nanocarrier for delivering a combination of anticancer drugs, cisplatin (Pt) and doxorubicin (DOX), to improve anticancer activity [72]. In this investigation, the authors successfully developed and characterized nano-sized pGO-Pt/DOX particles through various analytical techniques, such as zeta-potential, TEM, Raman, UV–vis, and FTIR. The results of the study indicated that the introduction of pGO enhanced the drug delivery efficacy of cisplatin (Pt). The optimized weight ratio of DOX: Pt: pGO was found to be 0.376:0.376:1. In vitro experiments demonstrated that pGO-Pt/DOX nanoparticles effectively entered tumor cells, leading to increased cell apoptosis and necrosis, resulting in higher growth inhibition compared to single drug delivery systems or free drugs. In vivo data showed reduced toxicity to normal organs with pGO-Pt/DOX, while tumor inhibition, histopathology observations, and immunohistochemical staining supported the superior anticancer effect of the dual-drug delivery system compared to free drugs.

The study performed by Wen et al. focused on addressing a significant challenge in the field of intracellular drug delivery using PEG-functionalized nano-graphene oxide (NGO) [45]. PEGylation is a widely employed strategy to enhance the stability and circulation time of nanocarriers, but it often hinders efficient drug release due to the PEG shell acting as a diffusion barrier. This study explored the development of a redox-responsive PEG detachment mechanism in a novel nano-graphene oxide construct, NGO-SS-mPEG, designed to overcome this issue. NGO-SS-mPEG was engineered to maintain stability and solubility in physiological environments while selectively detaching its PEG shell in response to elevated intracellular glutathione (GSH) levels. This controlled release of encapsulated drugs, such as doxorubicin hydrochloride (DXR), occurred rapidly and correlated with increased intracellular GSH concentrations. This design not only improves drug release efficiency but also offers a promising approach for tumor-selective drug delivery.

Another study focused on the development of (PEG) decorated graphene oxide GO nanosheets for controlled release of curcumin, an anti-cancer drug [60]. GO was synthesized using the Hummers chemical method and then conjugated with PEG using EDC/NHS catalysts to create GO-PEG. Curcumin was loaded onto GO-PEG, forming GO-PEG-Cur. The study found that 4.5% of curcumin was loaded onto GO-PEG and drug release rates were pH-dependent, with 50% release at pH 5.5 and 60% at pH 7.4 after 96 h. The nanocarrier displayed a zeta potential of −13.9 mV, indicating a negative surface charge that could potentially delay phagocytic activity in the bloodstream. These findings suggested that the developed nanocarrier is biocompatible and suitable for drug delivery systems, both in vitro and in vivo. The pH-dependent drug release is promising for targeted therapy, particularly for tumors with a slightly alkaline pH, offering a novel approach to controlled curcumin delivery.

In another study, a PEG-bis amine (PEGA) functionalized GO/iron oxide nanocomposite was synthesized to serve as a drug-loading platform, with methotrexate (MTX) as the model anticancer drug [61]. Cytotoxicity assays revealed higher toxicity against HeLa and MCF-7 cancer cell lines compared to free MTX, demonstrating the nanocarrier’s efficacy. Drug release studies indicated a first-order kinetics model, while blood compatibility tests affirmed the nanocarrier’s safety. The study also highlighted the advantages of this nanocomposite, including ease of synthesis, magnetic separation, PEGA modification for enhanced biocompatibility, and positive charge for improved cancer cell binding.

Yao et al. presented a comprehensive investigation into the development of a multifunctional GO drug carrier for the targeted delivery and controlled release of Dox into cancer cells, particularly hepatocarcinoma cells [73]. The synthesized GO-based carrier, denoted as GO/PEI.Ac-FI-PEG-LA, possessed several crucial features for effective drug delivery. The modification of GO with polyethyleneimine (PEI) and subsequent derivatization with fluorescein isothiocyanate (FI) and PEG-linked lactobionic acid (LA) allowed for the specific targeting of cancer cells that overexpress asialoglycoprotein (ASGPR) receptors. Notably, this carrier exhibited good water solubility and stability at a range of pH levels, ensuring its potential applicability under physiological conditions. The study achieved a high drug loading percentage (85%) and a pH-responsive controlled release of Dox, with a faster release rate at acidic pH levels, which is particularly relevant for cancer therapy. Furthermore, the GO/PEI.Ac-FI-PEG-LA/Dox carrier demonstrated good cell viability in tested concentrations and effectively inhibited the growth of cancer cells. The incorporation of LA-PEG groups enhances its targeting specificity, and the pH-responsive controlled release of Dox makes it a valuable tool in cancer treatment, potentially reducing side effects associated with drug toxicity.

A nanocarrier was synthesized by amidation, grafting folate-terminated PEG (FA–PEG–NH2) onto carboxylated GO nanosheets (CG) [74]. In terms of stability and compatibility, the CG–PEG–FA nanocarrier exhibited consistent dispersibility in PBS and displayed exceptional cytocompatibility, making it well-suited for biomedical applications. A notable feature of the nanocarrier was its high drug-loading capacity, which was achieved through π-π stacking interactions, showing the Dox loading with a capacity of 0.3993 mg. The nanocarrier also demonstrated a specific ability to target cells with folate receptors, a frequently used biomarker element for cancer therapy. Moreover, the nanocarrier showed pH-responsive controlled drug release properties, allowing for the precise delivery of drugs to cancer cells and potentially reducing side effects.

Jeshvaghani et al. introduced a pH-responsive, sustained-release nanocomposite composed of PEG, GO, and natural silk fibroin (SF) protein [62]. Using a double nano-emulsification method with sweet almond oil as the organic phase, the study aimed to fabricate a biocompatible and targeted pH-sensitive drug delivery system to improve the efficacy of the anticancer drug Dox. The nanocarrier demonstrated a cumulative release percentage of 95.75%, with a preference for acidic environments. This suggested a dissolution-controlled anomalous release mechanism at pH 7.4 and a diffusion-controlled anomalous mechanism at an acidic pH. Furthermore, the nanocomposite exhibited increased toxicity and apoptotic cell death in MCF-7 cancer cells compared to free DOX.

Yang et al. demonstrated loading of FAM-cDNA21 and Dox onto the GO surface, followed by their efficient uptake by cancer cells [75]. In the acidic lysosome environment, Dox was released and exerted its anticancer effects, while FAM-cDNA21’s interaction with miR-21 led to miR-21 silencing. This innovative approach allowed for the collaborative action of Dox and cDNA21, even at lower Dox dosages, potentially paving the way for reduced toxicity in cancer therapy. The Dox-GO-cDNA21 system is shown to be effective in delivering anticancer drugs and nucleic acids into cancer cells, reducing the required Dox dosage by approximately half without compromising efficacy.

A study conducted by Yu et al. addressed the challenge of treating depression, a chronic mental disorder with significant health implications, largely due to the restrictive nature of the blood–brain barrier (BBB) [76]. This barrier limits the distribution of antidepressant drugs within the brain. In response, the authors developed a novel brain-targeted drug delivery system using borneol-modified PEGylated GO (GO-PEG-BO). GO-PEG-BO demonstrated excellent biocompatibility and the capacity to penetrate the BBB effectively. This penetration was achieved through the opening of tight junctions and the inhibition of the BBB efflux system. GO-PEG-BO exhibited targeted distribution in the brain as demonstrated by in vivo studies, signifying its potential as a brain-targeted drug delivery system. Another study described the synthesis of a novel graphene-based nanocomposite, GO-PEG-FeOOH, by in situ growth of hydrous ferric oxide (FeOOH) nanorods on PEG modified GO sheets [77]. This innovative material exhibited impressive albumin adsorption capacity of 1377.4 mg/g for BSA. The selective adsorption of albumin is primarily attributed to strong hydrogen bonding interactions between the FeOOH nanorods and the albumin, leading to superior selectivity compared to other graphene-based materials.

A recent study by Demirel et al. showed efficient delivery of Dox and anti-GAPDH siRNA via PEG-conjugated reduced GO. An azide functionality containing poly[(poly(ethylene glycol) methyl ether methacrylate)-co-(3-azidopropyl methacrylate)-co-(methyl methacrylate)-co-(1-pyrenemethyl methacrylate)] (P(PEGMA-co-AzPMA-co-MMA-co-PMA), PAMP) copolymer was synthesized, and this copolymer was conjugated on the surface of rGO (PAMP-CP-rGO) for controlled release of dual-loaded nanocomposite. This dual-loaded nanocomposite showed high transfection efficiency and selective toxicity against cancer cells only, making it a safe and adaptable delivery platform [78].

Islam et al. synthesized a nanodrug delivery system by loading podophyllotoxin onto a GO-PEG surface (GO/PEG/PTOX) with a 25% loading ratio and 50% drug release after 48 h. It was demonstrated that GO/PEG/PTOX nanocomposite can be applicable in type-2 diabetes mellitus treatment as it efficiently inhibited α-amylase and α-glucosidase, exhibiting IC_50_ values of 7 and 5 mg/mL, respectively. Notably, these values closely align with the IC_50_ observed for pure PTOX [79].

Hu et al. synthesized a nanocomposite where PEG-GO was conjugated with lectin protein-Con A and pesticide epoxiconazole (EPX) to increase the antifungal activity of EPX against spore germination in Magnaporthe oryzae. This GO-PEG-ConA nanocomposite inhibited the spore germination in M. oryzae by 41.7% suggesting its potential role in controlling rice blast lesions in rice seedlings [65].

## 4. PEG-Engrafted GO for Enhancement of Nucleic Acid Amplification

Nucleic acid amplification technologies (NAATs) such as polymerase chain reaction (PCR), quantitative real time PCR (qPCR), and loop-mediated isothermal amplification (LAMP) have revolutionized molecular diagnostics by enabling easy, sensitive, specific, and high-throughput detection of a wide range of infectious diseases [80,81]. Our group has reported the beneficial effects of poly(ethylene glycol)-engrafted nanosized GO (PEG-nGO) in the improvement of NAATs like PCR, qPCR, and LAMP.

### 4.1. Polymerase Chain Reaction

**Figure 10:**
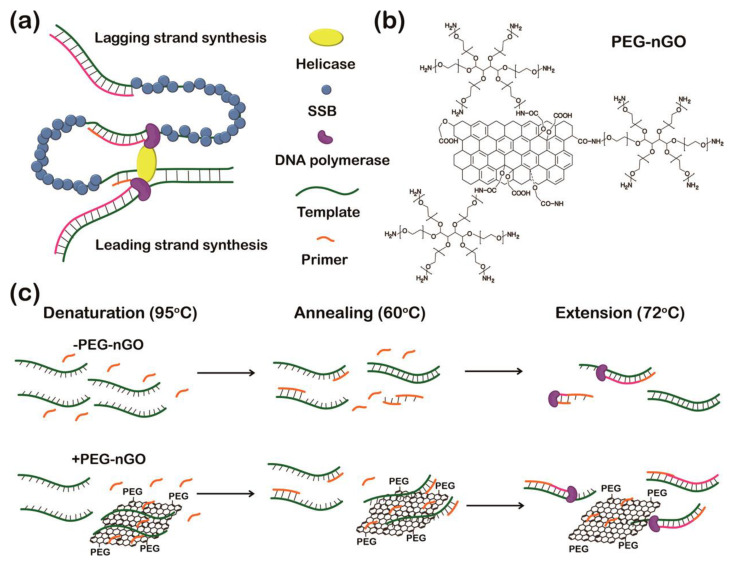
Enhancement of PCR by PEG-nGO. (**a**) PEG-nGO analogous to SSB as an inhibitor of strand reannealing in DNA replication. (**b**) Structure of PEG-nGO. (**c**) Schematic representation of PCR facilitation by PEG-nGO by adsorbing excess primer, thus reducing primer dimerization and false priming. Reprinted with permission from [63]. Copyright © 2016, American Chemical Society.

PCR is a synthetic amplification technique that offers extensive utility in diagnostics and molecular biology [82,83] but is limited by unintended reannealing of DNA, such as primer dimer formation and erroneous priming, resulting in compromised specificity [84,85]. Our group explored whether PEG-nGO enhances the performance of PCR by adsorbing excess primers during the PCR process [63]. During the initial phase of PCR, surplus primers bind to PEG-nGO and are subsequently released as PCR progresses, which aids in the reduction of primer dimer formation and erroneous priming. It has been hypothesized that GO can mimic the role of single-stranded DNA binding proteins due to its ability to bind to single-stranded DNA, which might prevent denatured single strands of template DNA from reannealing during replication (Figure 10a). However, direct utilization of GO in PCR is often limited due to its insolubility in high salt concentrations [86] (typical in PCR mixtures) and its ability to adsorb proteins [87,88] (e.g., polymerase) through noncovalent interactions. To address these limitations, PEG was conjugated onto the surface of nanosized GO (Figure 10b), which increases the solubility of GO in PCR mixtures containing high salt concentrations (Mg^2+^) and reduces the non-specific binding of Taq DNA polymerase onto the GO surface, allowing unhampered activity of Taq DNA polymerase [63]. It was conjectured that in the early stage of PCR, PEG-nGO can impede the formation of primer dimers by absorbing excess primers and inhibiting the reannealing of DNA strands in later PCR cycles by adsorbing the amplified DNAs (Figure 10c).

PCR amplification of the target gene was carried out in the presence of three carbon composites (GO, nanosized GO (nGO), and PEG-nGO) and interestingly, a specific PCR-amplified band was only observed in the presence of PEG-nGO. This intriguing observation prompted us to investigate the affinity of PCR components such as ssDNA and Taq DNA polymerase with these three carbon composites. GO and nGO exhibited stronger binding affinities towards single-stranded DNA than PEG-nGO, which indicated that the weaker binding affinity of PEG-nGO towards single-stranded DNA is more suitable for PCR reactions. Furthermore, Taq DNA polymerase was found to bind to GO and nGO, leading to inhibited enzymatic activity, whereas PEG-nGO did not bind to Taq DNA polymerase, preserving its enzymatic activity. We evaluated the impact of PEG-nGO on multiple rounds of PCR since they are prone to produce non-specific amplicons due to the higher concentration of amplified DNAs. In the consecutive rounds of PCR, only the target DNA bands were observed in the presence of PEG-nGO without any smearing, whereas nonspecific smeared bands were observed in the absence of PEG-nGO. Next, to assess the effect of PEG-nGO on primer dimer formation, primers were added to the PCR mixture without any template. In the absence of PEG-nGO, bands indicating dimerized primers were observed, but these dimerized primer bands disappeared when PEG-nGO was added to the PCR mixture. PEG-nGO also demonstrated reduced mispairing between template DNA and primers at low annealing temperatures by adsorbing excess primers on its surface, thereby enhancing the specificity of PCR. This study demonstrated that PEG-nGO significantly enhanced the specificity and efficiency of PCR by adsorbing excess primers in the initial stage of PCR, which resulted in reduced primer dimerization and prevented reannealing of amplified DNA in later stages of PCR, facilitating primer annealing to template strands.

### 4.2. Quantitative Real-Time Polymerase Chain Reaction

Encouraged by the beneficial effect of PEG-nGO on end-point PCR assay, our group expanded the applicability of PEG-nGO by investigating the effect of PEG-nGO on quantitative fluorescence-based qPCR assay [64]. qPCR is the most availed tool in clinical diagnostics that offers the advantage of real-time detection of nucleic acid during the amplification process, eliminating the need for post-amplification gel electrophoresis for amplicon detection [89,90]. qPCR-based amplification is either detected by dsDNA binding dyes or fluorogenic probes where the fluorescence signal is directly proportional to the amount of amplification [91]. In the probe-based method, a specific probe with a fluorescent reporter and quencher emits fluorescence when the target is present, ensuring specificity, but it requires custom probe synthesis for each sample [92]. In dye-based methods, fluorescence signals correlate with the amount of amplified dsDNA, but they can generate false positives due to non-specific binding of dyes to dsDNA [93]. Since qPCR is regarded as the gold standard in diagnostics, addressing the limitations of qPCR, such as nonspecific amplification and false positive/false negative signals, is essential for specific detection of diseases.

**Figure 11:**
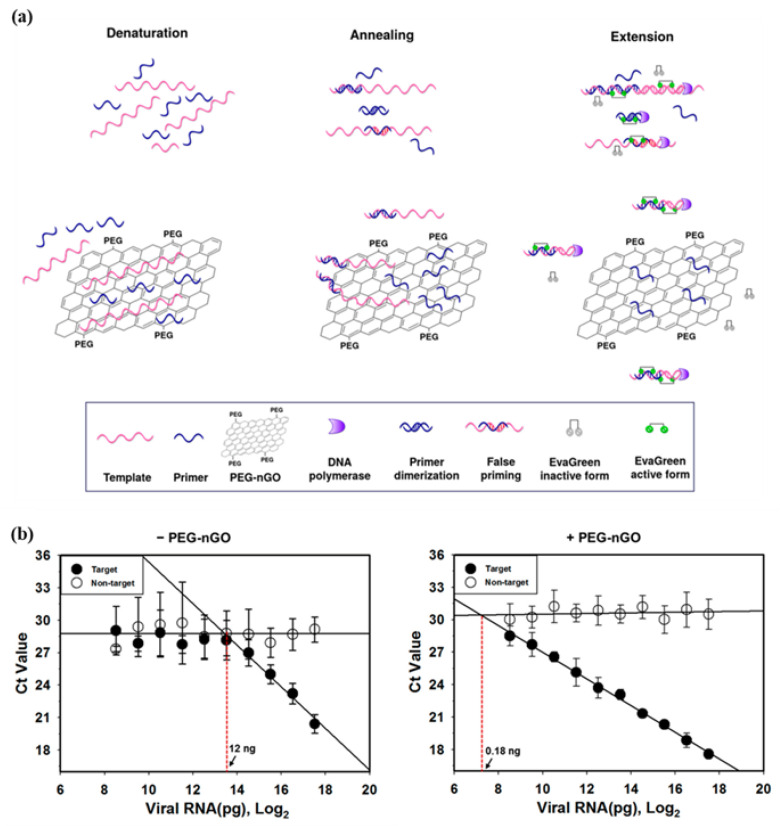
Facilitation of qPCR by PEG-nGO. (**a**) Schematic representation of dye-based PENGO-qPCR. (**b**) Effect of PEG-nGO on the sensitivity of qPCR. The presence of PEG-nGO enabled the distinction between the target and non-target even at low template concentration. Reprinted from [64].

To explore the hypothesis that PEG-nGO can enhance the specificity of qPCR by adsorbing excess primers in the initial phase of qPCR, thus reducing primer dimerization and false priming, we utilized dsDNA dye-based qPCR to detect influenza viral RNA as a model system (Figure 11a). We compared two commonly used dsDNA binding dyes, SYBR Green I (SGI) and EvaGreen (EG) in PEG-nGO-based qPCR (PENGO-qPCR), and found that EV was suitable for PENGO-qPCR due to reduced adsorption of EG-dye-dsDNA complex on the surface of PEG-nGO. PENGO-qPCR demonstrated improved specificity as compared to conventional qPCR by exhibiting a higher melting temperature difference between the target and non-target, as well as a lower Ct value for the target than the non-target. Also, PENGO-qPCR was found to be 67-fold more sensitive than conventional qPCR, enabling differentiation between the target and non-target at low nucleic acid concentrations as well (Figure 11b). This study demonstrated that PEG-nGO diminishes nonspecific amplification such as false priming and primer dimerization in dye-based qPCR, making it suitable for specific and sensitive gene detection.

### 4.3. Loop-Mediated Isothermal Amplification

Loop-mediated isothermal amplification (LAMP) is a rapid and robust NAAT which is carried out at isothermal temperature, thus eliminating the need for a thermocycler [94,95,96]. LAMP utilizes 4–6 primer sets to create foldback structures, followed by their elongation using Bacillus stearothermophilus DNA polymerase, facilitated by strand displacement activity [97,98]. LAMP reactions often produce unintended non-specific products due to primer dimerization and mismatched hybridization as LAMP employs multiple primers with high concentration [99,100]. Fluorescence enhancement attributed by dsDNA-binding dyes intercalating with any dsDNA makes it challenging to differentiate between target and non-target samples, potentially resulting in false positives [101].

**Figure 12:**
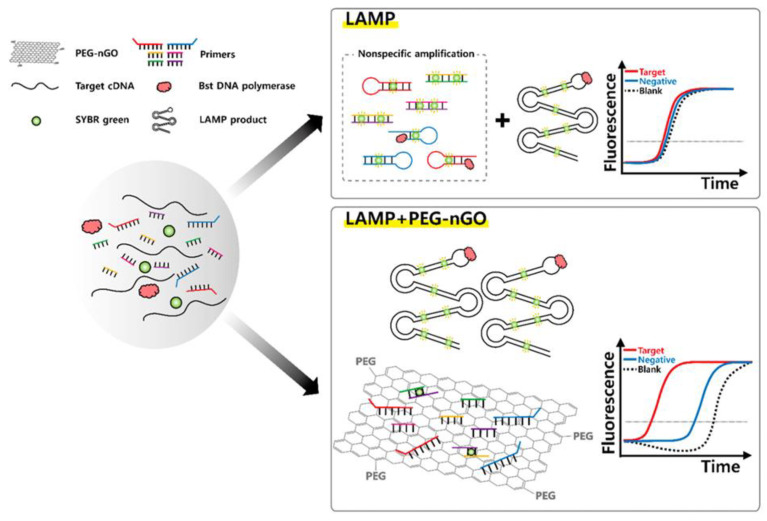
Schematic representation of the enhancement of specificity in LAMP by PEG-nGO. Reprinted from [102].

Motivated by PEG-nGO-based enhanced specificity in PCR, we investigated its potential in LAMP, aiming to exploit its ssDNA adsorption abilities with multiple ssDNA primers [102]. We tested PEG-nGO in the LAMP assay for hepatitis C virus (HCV) gene detection and found that it effectively prevented non-specific amplification by adsorbing excess ssDNA primers, resulting in reduced background fluorescence (Figure 12). We observed that inclusion of PEG-nGO notably improved the specificity and sensitivity of LAMP assay by increasing the difference in fluorescence signals between target and non-target samples. We further explored the applicability of PEG-nGO in clinical diagnostics by applying PEG-nGO-based LAMP for the detection of multiple HCV clinical samples derived from human serum. PEG-nGO-based LAMP was found to improve false-positive detection 1.75 times more than traditional LAMP, enhancing target DNA amplification while reducing background signals through excess primer adsorption. Thus, PEG-nGO-based LAMP is suitable for precise target gene detection with high sensitivity and specificity.

## 5. Future Perspective

GO has firmly established itself as a revolutionary material with significant implications in the field of biomedicine. Its distinctive characteristics, such as its large surface area, facile surface modification, hydrophilicity, moderate electrical conductivity, robust mechanical properties, and ability to bind with a variety of biomolecules have proven greatly beneficial in advancing drug delivery systems and biosensors. Despite having all these advantageous properties, the utilization of GO is often limited to various biomedical applications. These limitations include poor solubility, cytotoxicity, generation of immune responses, nonspecific binding of various biomolecules on its surface, and strong binding affinity to nucleic acids. To overcome these limitations, GO can be easily conjugated with biocompatible polymer PEG, due to its larger surface area and presence of various oxygen containing functional groups.

PEGylation of GO offers several advantages such as imparting adequate solubility and stability in various physiological media, minimized toxicity, specific binding of biomolecules, enhanced retention time of drugs, and weaker binding affinity of nucleic acid on its surface. Thus, PEG-conjugated GO not only retains the beneficial properties of GO but also effectively addresses its limitations, positioning it as a favorable option for a wide array of biomedical applications. Therefore, PEGylated GO has the potential to aid the development of therapeutic solutions in the biomedical field.

## Figures and Tables

**Table 1:** PEG-engrafted GO via covalent conjugation for various biomedical applications.

PEG-GO Composite	Biomolecule Delivered	Conjugation Agent for PEG	Application	Reference
PEG-nGO	PNA	Chloroacetic acid, EDC	Reduced cytotoxicity and improved solubility	[54]
PEG-RGO	ssDNA	EDC	Enhanced solubility	[55]
GO-PEG-R8	siRNA & plasmid DNA	EDC, NHS	Biocompatibility and high loading capacity	[56]
NGO-PEG-PEI	Plasmid DNA	EDC	Reduced cytotoxicity	[57]
GO-PEG/PTX	Paclitaxel	EDC, NHS	High stability and reduced toxicity	[47]
Ce6/Dox/pGO	Photosensitizer chlorin e6 & Dox	EDC	Enhanced solubility and biocompatibility	[58]
GO-PEG_10K-6arm_	Oridonin and MTX	NaOH, EDC, NHS	Low cytotoxicity and high loading capacity	[59]
GO-PEG-Cur	Curcumin	NaOH	Controlled drug release	[60]
PEGA	MTX	FeCl_3_·6H_2_O, FeCl_2_·4H_2_O	Enhanced biocompatibility and increased circulation time	[61]
PEG/GO/SF	Silk fibroin & Dox	Acetic acid	Enhanced stability and solubility	[62]

## Data Availability

Not applicable.
